# Editorial: Women in experimental pharmacology and drug discovery 2023

**DOI:** 10.3389/fphar.2024.1439405

**Published:** 2024-07-12

**Authors:** Patricia Rijo, Emel Timucin, Maria M. M. Santos, Valeria Bruno

**Affiliations:** ^1^ CBIOS—Lusófona University’s Research Center for Biosciences and Health Technologies, Lisbon, Portugal; ^2^ Instituto de Investigação do Medicamento (iMed.ULisboa), Faculdade de Farmácia, Universidade de Lisboa, Lisbon, Portugal; ^3^ Department of Biostatistics and Medical Informatics, School of Medicine, Acibadem University, Istanbul, Türkiye; ^4^ IRCCS Neuromed, Pozzilli, Italy; ^5^ Department of Physiology and Pharmacology, University Sapienza, Rome, Italy

**Keywords:** natural products, sustainable ingredients, therapeutic potential, antioxidative components, pharmacology

As editors of Frontiers in Pharmacology, we are thrilled to introduce the Research Topic “*Women in Experimental Pharmacology and Drug Discovery 2023*,” marking a significant part of our series commemorating International Women’s Day on 8 March 2023. This Research Topic serves as a testament to the remarkable achievements of women scientists in pharmacology, spotlighting their diverse and impactful research endeavors. From pioneering advancements in theory and experimentation to developing innovative methodologies, these contributions address pressing challenges and propel the field forward.

According to UNESCO, less than 30% of researchers worldwide are women. Persistent biases and gender stereotypes deter many girls and women from pursuing careers in science-related fields, particularly in STEM. However, science and gender equality are essential for sustainable development. To change traditional mindsets, it is crucial to promote gender equality, defeat stereotypes, and encourage girls and women to pursue STEM careers.

Frontiers in Pharmacology is proud to offer this platform to highlight the outstanding work of women scientists. Here are some key studies featured:

The study, led by Kamel et al., investigates the hepatoprotective potential of Ocimum sanctum L. (Tulsi) against galactosamine-induced toxicity in rats, identifying and quantifying bioactive compounds such as rutin, ellagic acid, and quercetin, which demonstrate significant protective effects.

The study, led by Phongpradist et al., examines the *in vitro* effects of black soldier fly larvae (BSFL) oil, highlighting its potential as a sustainable, high-functional ingredient for inhibiting hyaluronidase, providing antioxidant benefits, promoting skin whitening, and offering UVB protection, with significant findings showing its efficacy comparable to well-known cosmetic ingredients.

The study, led by Liang et al., explores the antioxidative and anti-hyperuricemic components of Rodgersia podophylla A. Gray, identifying potent bioactive compounds such as norbergenin, catechin, procyanidin B2, and gallic acid through bio-affinity ultrafiltration with superoxide dismutase (SOD) and xanthine oxidase (XOD), demonstrating significant antioxidant and enzyme inhibition activities.

In their review, Collotta et al. delve into antisense oligonucleotides (ASOs), synthetic RNA or DNA molecules that target disease-causing genes. ASOs offer a novel therapeutic strategy, particularly for orphan genetic disorders. The review outlines advancements addressing pharmacological challenges and provides a comparative analysis of ASOs approved by regulatory agencies, showcasing their clinical potential.

The study by Koni et al. investigates the role of CXCL17 in lung adenocarcinoma, showing that overexpression of CXCL17 in A549 cells significantly enhances their migration and invasion capabilities without affecting cell proliferation, suggesting a potential role of CXCL17 in promoting tumor metastasis.

Sharma et al. explored the anti-rheumatic potential of Nyctanthes arbor-tristis (NAT) extracts. Their study demonstrated the safety and efficacy of a 500 mg/kg dose of NAT leaf extract in rats, with significant reductions in inflammation and improvements in arthritis symptoms. NAT shows promise as a therapeutic option for inflammatory arthritis.

Inselman et al. investigated how black cohosh extract and risedronate interact in improving bone health in ovariectomized rats. They found that while high-dose risedronate significantly increased bone mineral density (BMD) in the femur and vertebrae, black cohosh extract had minimal effect alone and did not interfere with risedronate’s BMD-enhancing properties when co-administered.

Yavuz et al. examined the effects of social isolation on mental health and cognition in Sprague Dawley (SD) and Wistar Albino (WIS) rats, amid the COVID-19 pandemic. They found that while SD rats learned faster, they were more prone to depression after isolation compared to WIS rats. Elevated levels of certain trace elements in isolated SD rats hint at their role in stress responses, offering insights for future preventive treatments against stress-related neurobehavioral issues.

Öz Arslan et al. reviewed the potential of orphan G protein-coupled receptors (GPCRs) in treating neurodegenerative diseases like Alzheimer’s, Parkinson’s, Huntington’s, and multiple sclerosis. Despite limited therapies, orphan receptors offer promising targets due to their roles in critical cellular processes. The review discusses their therapeutic potential, recent advances in drug discovery, and outlines future research directions and challenges.

Zhao et al. reviewed the link between adiponectin and endometriosis. Adiponectin, primarily from adipose tissue, influences energy regulation and estrogen-related diseases. Despite evidence suggesting lower adiponectin levels in endometriosis patients, its exact role remains unclear. The review highlights adiponectin’s involvement in key biological processes related to endometriosis and emphasizes the need for further research to identify therapeutic targets and understand the condition better.

Promoting gender equality in pharmacology is essential for scientific advancement and sustainable development. The diverse research presented here underscores the vital contributions of women scientists. By supporting and celebrating their work, we foster a more inclusive and innovative scientific community.

Frontiers in Pharmacology remains dedicated to this mission, showcasing the remarkable work of women in the field and inspiring future generations.

